# Root Foraging Performance and Life-History Traits

**DOI:** 10.3389/fpls.2016.00779

**Published:** 2016-06-09

**Authors:** Martin Weiser, Tomáš Koubek, Tomáš Herben

**Affiliations:** ^1^Department of Botany, Faculty of Science, Charles University in PraguePrague, Czech Republic; ^2^Institute of Botany of the ASCRPrůhonice, Czech Republic

**Keywords:** root foraging, clonal plants, phenotypic plasticity, plant development and life-history traits, plant–soil (below-ground) interactions, specific leaf area, soil heterogeneity, vegetative reproduction

## Abstract

Plants use their roots to forage for nutrients in heterogeneous soil environments, but different plant species vastly differ in the intensity of foraging they perform. This diversity suggests the existence of constraints on foraging at the species level. We therefore examined the relationships between the intensity of root foraging and plant body traits across species in order to estimate the degree of coordination between plant body traits and root foraging as a form of plant behavior. We cultivated 37 perennial herbaceous Central European species from open terrestrial habitats in pots with three different spatial gradients of nutrient availability (steep, shallow, and no gradient). We assessed the intensity of foraging as differences in root placement inside pots with and without a spatial gradient of resource supply. For the same set of species, we retrieved data about body traits from available databases: maximum height at maturity, mean area of leaf, specific leaf area, shoot lifespan, ability to self-propagate clonally, maximal lateral spread (in clonal plants only), realized vegetative growth in cultivation, and realized seed regeneration in cultivation. Clonal plants and plants with extensive vegetative growth showed considerably weaker foraging than their non-clonal or slow-growing counterparts. There was no phylogenetic signal in the amount of expressed root foraging intensity. Since clonal plants foraged less than non-clonals and foraging intensity did not seem to be correlated with species phylogeny, we hypothesize that clonal growth itself (i.e., the ability to develop at least partly self-sustaining ramets) may be an answer to soil heterogeneity. Whereas unitary plants use roots as organs specialized for both resource acquisition and transport to overcome spatial heterogeneity in resource supply, clonal plants separate these two functions. Becoming a clonal plant allows higher specialization at the organ level, since a typical clonal plant can be viewed as a network of self-sustainable harvesting units connected together with specialized high-throughput connection organs. This may be an effective alternative for coping with spatial heterogeneity in resource availability.

## Introduction

For plants, soil is the source of various essential resources with contrasting repletion and depletion dynamics and spatial patterns (Craine and Dybzinski, 2013). Phenotypic plasticity in root growth, architecture, and spatial placement may be an answer to the soil heterogeneity and low predictability (Bradshaw, 1965). Ample evidence of a plastic response in root growth and placement has been obtained from experimental systems illustrating root searching patterns in response to gradients of water and nutrients (Drew, 1975; Hodge et al., 1999). Indeed, roots are the plant organ for which foraging for resources has been most convincingly demonstrated (Hutchings and de Kroon, 1994). However, the degree of such root plasticity strongly differs among species (e.g., Campbell et al., 1991; Kembel and Cahill, 2005; Keser et al., 2015), indicating the existence of a factor that constrains this potential. We see two possible sources of such constraint: (i) differences in growth rate and resulting overall root system size, and (ii) differences in the processes that determine the size-independent component of root system shape.

Differences among individuals in growth rate and overall root system size constitute their passive plasticity. In contrast, differences in the processes that determine the size-independent component of root system shape, and result into change in allometric patterns of the body form, constitute active plasticity. It typically occurs as a result of responsive behavior to an environmental signal. A common example of this is root foraging, that is, changes in spatial root system allocation in response to a nutrient or water gradient (Hutchings and de Kroon, 1994; McNickle and Brown, 2014b). Although both types of response are based on growth, passive plasticity and active plasticity are in principle independent of each other. Only passive plasticity is the direct outcome of overall amount of growth (Weiner, 2004; Van Kleunen and Fischer, 2005). However, it may constrain options available for active plasticity to take place.

Growth and the resulting size patterns constitute one of the most fundamental ecological differences (Gaudet and Keddy, 1988; Cornwell et al., 2014; Aarssen, 2015). With an increasing rate of root growth, root-perceived spatial heterogeneity naturally decreases. The faster the root tip moves through the soil matrix, temporal heterogeneity it processes decreases in grain size. Root plasticity cannot reflect soil heterogeneity if heterogeneity occurs on too small a scale (Alpert and Simms, 2002), but scale size is determined by traits of the individual, namely, size and growth rate. As plant species differ in their growth rate even within a single environment (Grime and Hunt, 1975), differences between fast and slow species may open the field for root systems specialized in harvesting small-scale patches at the level of heterogeneity overlooked by fast-growing species.

Passive plasticity is very unlikely to be the only factor underlying interspecific differences in root system plasticity, namely, root foraging. If root system plasticity is more pronounced in fast-growing species simply because of multiplicative growth effects (Aanderud et al., 2003; Kembel and Cahill, 2005), it would result in a paradox: slow species should forage better, that is, should be more plastic, to respond to rich patch presence at the small scale, but cannot, whereas fast-growing species could be more plastic, but need not to forage, as they average the outcome of resource acquisition across the patches and grow through the rich patches (Alpert and Simms, 2002). The solution to this paradox involves an actively plastic element of behavior that is (or at least can be) more pronounced in slow species.

Diversity in root plastic responses to various cues [e.g., nutrient pre-emption (Padilla et al., 2013), root overproliferation (Gersani et al., 2001), patch avoidance (Semchenko et al., 2007; McNickle and Brown, 2014a)] implies that apart from the growth rate, there may be several life-history traits (body constraints) that constrain the plastic response of root systems to soil heterogeneity. However, the identity of these constrains is largely unknown. Jansen et al. (2005) found negative correlation of root foraging precision, and rather complex trait, species ability to withstand flooding. Combining root foraging precision data (Johnson and Biondini, 2001) with root and leaf ecophysiological traits (Tjoelker et al., 2005), Kembel et al. (2008) found positive correlation of root foraging precision and root and leaf nitrogen content, and negative correlation of the root foraging precision with root longevity.

Constraints shaping plant shoot plasticity are better known. Because root and shoot plasticity have been found to be correlated (Grime et al., 1997), we may expect that the same or similar life-history traits could be used equally well to predict root plasticity. For example, growth form and clonality constrain the plasticity of plant shoots (Dong and de Kroon, 1994; Huber, 1996; Huber et al., 1999). Moreover, the existing knowledge of ecological functions of life-history traits (Westoby, 1998; Cornwell et al., 2014) may help to identify potential constraints/predictors of passive and active plasticity. Some traits are likely to determine interspecific differences in size and growth (i.e., components of passive plasticity). In particular, specific leaf area and maximum height are known to be good proxies of size and growth rate differences and hence clear candidates for the examined pool of potentially constraining traits. Constraints on active plasticity are more elusive, but should be searched for among traits that constrain size-independent differences in plant bodies. Among them, traits that moderate reproductive effort are crucial for local dominance and species coexistence (Herben et al., 2014, 2015; Klimešová et al., 2016). Namely, seed reproduction rate, vegetative reproduction rate, clonality (together with lateral spread for clonal species) and shoot longevity describe the reproductive process well, because these traits describe both the reproductive outcome and its dynamics.

A great advantage of all these life-history traits is that their values are known for large sets of species. Either these traits are already cataloged in species descriptions (e.g., species height at maturity, clonality, leaf size) or may be easily obtained from species collections such as those in botanical gardens. Therefore, they can potentially be used as proxies for the ability of species to respond to below-ground heterogeneity which is much more difficult to measure.

In the work described in this article, we determined differences in root system plasticity in a large set of herbaceous species and examined potential constraints and predictors of these differences. To obtain root system plasticity estimates comparable to those in previous studies (e.g., Campbell and Grime, 1989; Campbell et al., 1991), we essentially replicated the approach based on root foraging, that is, allocation of roots in a patchy environment. We linked these estimates with life-history species traits that presumably predict either growth rate and size or size-independent differences in plant bodies. In addition, we also used realized vegetative and seed reproduction rates as estimates of the functional outcome of these traits (Herben et al., 2012). We compared the predictive power of these life-history traits with that of specific leaf area (SLA), the prominent trait reported to determine root system plasticity earlier (Grime et al., 1997).

## Materials and Methods

### Species Selection

We selected 43 herbaceous species from 14 families of the flora of the Czech Republic using the following criteria: (i) perennial hemicryptophyte growth form and (ii) occurrence in mesic unshaded or moderately shaded habitats [Ellenberg indicator value (EIV) for moisture <9 and EIV for light >5 (Ellenberg, 1992)]. We avoided species known for their taxonomic complexity. To represent the taxonomic composition of the flora, we used several species from four widespread families (Asteraceae, Caryophyllaceae, Poaceae, and Rosaceae), together with a few species from less diverse families. Ten species were tested in the year 2013; another 33 were tested in 2014. For the final analysis, we excluded 6 species because they had very small roots and, therefore, were vulnerable to errors in root biomass processing. See the final list of 37 species with additional information in **Supplementary Table S1** in Supporting Information.

### Species Life-History Trait Data

The following species life-history traits (hereinafter referred to as “traits”) were selected from several databases to represent the size and growth dynamics of the species involved: plant height at maturity, mean area of leaf, specific leaf area, shoot life span [cyclicity, see Klimešová and de Bello, 2009], clonality (i.e., capacity to form new ramets by clonal growth, a binary trait), and lateral spread (in meters) (J. Klimešová, unpublished data; Klimešová and de Bello, 2009). Values of these traits were taken from the LEDA trait base (Kleyer et al., 2008), CLO-PLA database Version 3.3 (Klimešová and de Bello, 2009) and (Kubát et al., 2002). Further, we used capacity for vegetative and sexual reproduction assessed by long-term observation in a botanical garden (Herben et al., 2012) as additional information on species reproductive strategy. These data correspond to the need for thinning performed by the gardener in order to balance species expansion (ordinal scale, 1..5). Mean leaf area was log-transformed before analysis. Some trait values were defined only for subsets of species, for example, lateral spread data were defined only for clonal species, whereas other trait values were simply unavailable in the databases we used. Missing values of both types were not included in the calculation of species trait–foraging ability correlations (see below). The species trait correlation matrix did not show high levels of collinearity (see **Supplementary Table S2**). Data on species phylogeny are taken from Daphne phylogeny (Durka and Michalski, 2012).

### Experimental Setup

The species were obtained as seeds from a commercial supplier (Planta Naturalis)^1^. Seeds were sown into seeding trays with clean sand in a greenhouse in June 2013 and the end of May 2014. All plants germinated within 1 month from sowing and were planted in August 2013 (July 2014) by species in a time sequence that spanned 2 weeks. We did this for two reasons: (i) to start with each species at approximately the same size and (ii) to spread the harvest period. The plants were planted into round 3-L pots (TEKU Pöppelmann MCI 19, inner top diameter 19 cm, bottom diameter 16 cm) with washed sand. The sand was washed with tap water in small batches in a concrete mixer until the water was clear. We took extra care to place the plant in the middle of the pot. Pots were placed on water-leveled perforated plates to avoid uneven mixing and leaks into other pots. Each pot was drip irrigated from two sides, and all pots received the same amount of fertilizer in the water. The treatments were created by changing the proportion of fertilizer in the drippers. There were three treatments: (i) control (no contrast, 2:2), (ii) low contrast (3:1), and (iii) high contrast (4:0). The precise dosage was dispensed by a mechanical dosing system (Dosatron, D25RE2). The commercial fertilizer was Wuxal Super (NPK 8:8:6 + micronutrients, Aglukon). We used the recommended dilution for adult plants (0.2%) as the maximum by diluting 10% stock fertilizer to 2%; the other concentrations were mixed similarly by diluting to 1.5, 1, and 0.5% of the stock. So we achieved final concentrations at the levels of 0.2, 0.15, 0.1, and 0.05% of the original fertilizer concentration.

The plants were harvested after 5 weeks after the transplant from seedling trays to pots, i.e., at the age of about 9 weeks, in the same sequence as they were planted. At this time, the roots of the fastest growing species almost reached pot walls and individuals of most of the species reached the size of fully grown plants. Each pot was divided into two halves in the middle of the plant’s rooting point by a sharpened iron sheet. Both halves of the pot were washed in water on a fine sieve, and all roots were extracted. The roots were dried at 65°C and weighted. These data are available as Dryad repository item (Weiser et al., 2016).

### Data Analysis

For each pot, we calculated the natural logarithm of root weight in each half of the pot and expressed root placement pattern as log (root quantity in nutrient-rich half/root quantity in nutrient-poor half). Logarithmic transformation effectively removes linear effect of plant size; that is, the values obtained are likely to express effects that are independent of it (active plasticity). For control pots with no contrast, instead of nutrient-poor and nutrient-rich halves, we used (arbitrarily chosen) the left and right halves of the pot. Hereafter we call this parameter “precision.”

Even in control pots, the balance data per species per treatment exhibited substantial skewness, as measured with the robust *medcouple* method [package *robustbase*, version 0.8-1-1, (Rousseeuw et al., 2012)]. Therefore, we used medians to represent species by treatment response and used non-parametric methods in species response estimation.

To assess whether the treatments used were effective in eliciting a root allocation response, we compared precision data for the control with data for low-contrast treatment and data for high-contrast treatment. Comparisons were done pairwise according to species identity, using the Wilcoxon test as implemented in *wilcox.test* procedure. Control data were used twice; therefore, we report the Bonferroni-corrected (multiplied by 2) *p*-values of these tests.

We obtained species-specific treatment effects—response as a shift in balance—through comparison of the precision values in the treated and control sets of pots. Specifically, we quantified them as a Mann–Whitney test statistics divided by the product of the numbers of individuals subjected to each treatment, $\frac{U}{\left(m\times n\right)},$ where *U* is the Mann–Whitney test score for difference in balance across contrasts (control versus high or low contrast), reported by *wilcox.test*; *m* is the number of control pots of the species; and *n* is the number of pots of the species subjected to low- or high-contrast treatment (Newcombe, 2006). These species-specific responses were centered to zero by subtracting 0.5.

In this way, we obtained two response parameters per species: one for precision difference between control and low contrast and the other for precision difference between control and high contrast. However, the species response estimates in low and high contrast correlated substantially; therefore we used only the high-contrast species response estimates for correlation with species traits. To calculate these correlations, we always used the non-transformed form of the parameter (“foraging”) and its absolute value (“plasticity”). The approach allowed us to take rich patch avoidance response into account as a part of the plastic reaction; partly discriminating between directional growth towards rich patches and directionless amount of plasticity.

The reliability of the response (i.e., stability of the difference between individuals from the control and contrast groups) was estimated by 1,000 bootstrap iterations on the data. In each iteration, both control and contrast balance values were bootstrapped. Because we did not assume any probability distribution for the balance differences, we used the ordinary bootstrap method, as implemented in the *boot* procedure [package *boot*, Version 1.3–5; (Davison and Hinkley, 1997; Canty and Ripley, 2015)].These stability measures were compared across contrasts with the Wilcoxon paired test, for which we used the difference between the 0.25 and 0.75 quantiles (i.e., “middle half”) of the bootstrapped values for each response estimate (high values mean low stability).

We correlated high-contrast response estimates in both forms with species traits using procedure *rcorr* (*Hmisc* package, Version 3.9–3; Harrell, 2012). For binary and ordinary traits (i.e., plant clonality, vegetative reproduction potential, generative reproduction potential, shoot longevity), we used Spearman’s correlation coefficient (ρ). We used Pearson’s correlation coefficient (*r*) for the remaining data. All analyzes were performed in the R statistical environment, Version 2.15.1 (R Core Team, 2012). To account for phylogenetic non-independence of species, we also performed phylogenetic regressions with high-contrast response estimates as dependent variables and individual traits as independent variables. Pagel’s lambda (transformation of branch lengths) were estimated by maximum likelihood analyzes with lambda set to one (Brownian motion evolution) were run as parallel checks, but their results are not reported due to low difference from the former. All phylogenetic regressions were performed using function *pgls* from the package *caper* (Orme et al., 2013) for R.

Phylogenetic conservatism of high-response estimates and of their absolute values was determined using Pagel’s lambda (Pagel, 1992). Maximum likelihood value of lambda was estimated using the function *pgls* from the package *caper* (Orme et al., 2013) for R.

## Results

**FIGURE 1:**
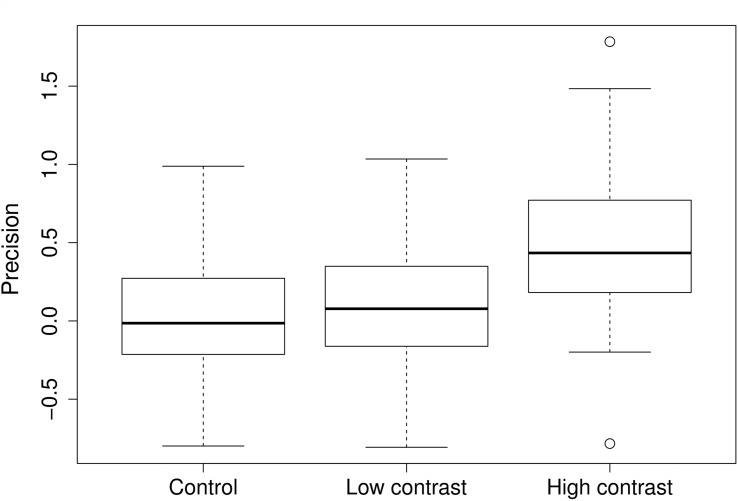
**Root placement in the control, low, and high nutrient contrast treatments.** Precision value is defined as natural logarithm of the ratio of the rich to the poor pot half. Zero value means equal amount of roots in both halves of the pot. Positive values mean more roots were placed into the nutrient rich half of the pot. Widths of the boxes show square-root of number of cases.

In general, high contrast elicited a substantial response in root allocation, whereas low contrast did not (**Figure 1**) (control vs. low contrast *V* = 299, *p* = 0.87; control vs. high contrast *V* = 120, *p* < 0.001).

**FIGURE 2:**
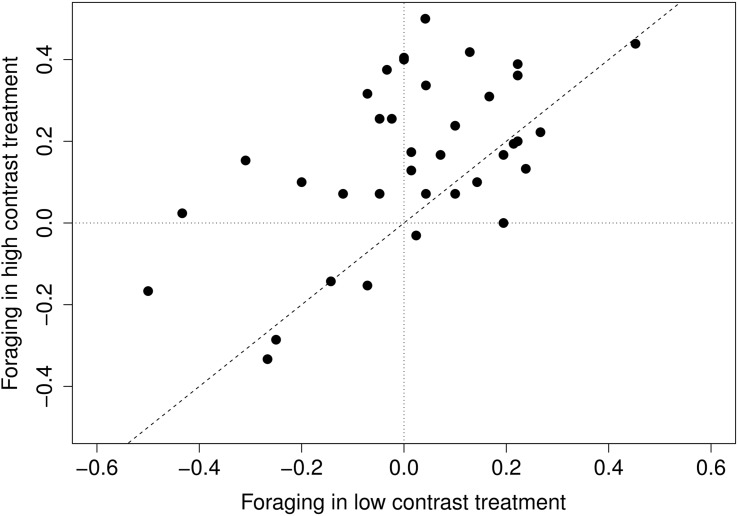
**Species response to different levels of contrast.** Low contrast foraging is the difference between low contrast and control treatment; high contrast foraging – is the difference between high contrast foraging and control treatment.

More species exerted a strong foraging response in high-contrast treatment than in low contrast treatment, but the responses under both treatments were substantially correlated to each other (Spearman’s ρ = 0.46, *p* = 0.004) (**Figure 2**; **Supplementary Figure S1**). Rather surprisingly, few species (*Bromus benekenii*, *Hypericum perforatum*, *Thalictrum lucidum*) avoided nutrient-rich patches in both contrast levels; their responses seemed to be quite stable. Response to the high-contrast treatment was significantly more stable (i.e., using bootstrap, we obtained a narrower set of the response estimate) than response to low-contrast treatment (*V* = 170.5, *p* = 0.018), but the stability of the responses within species across treatments did not correlate substantially (Spearman’s ρ = –0.24, *p* = 0.158).

Contrast values did not show any phylogenetic signal. Estimated confidence intervals of Pagel’s lambda for the high-contrast root foraging ability did not differ significantly from zero (confidence interval 0-0.31), while it was highly significantly different from 1 (Brownian motion evolution; *P* < 0.001, **Supplementary Figure S2**).

**FIGURE 3:**
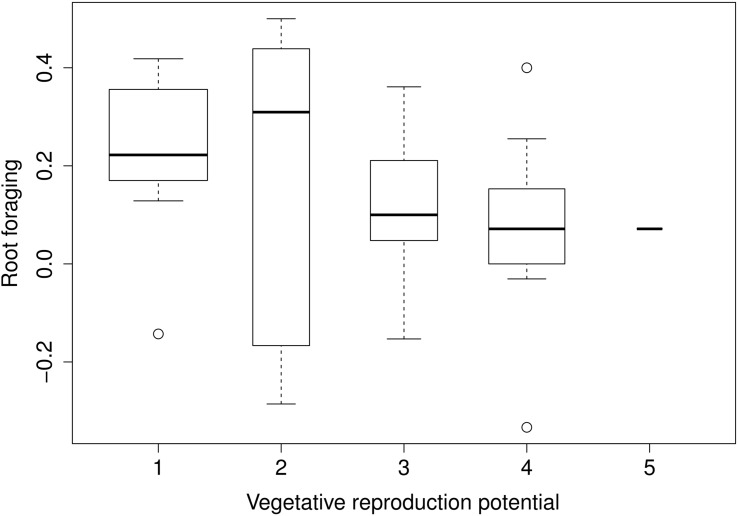
**Foraging estimates and vegetative reproduction potential.** [Vegetative reproduction potential: long-term ordinal scale data. Level of thinning needed in order to balance species expansion. Species scored at maximum level (5) must be thinned more than once a year, species at the minimum level (1) do not spread nor do not need thinning at all. See Herben et al. (2012) for further details explanation]. Widths of the boxes show square-root of number of cases (species).

**FIGURE 4:**
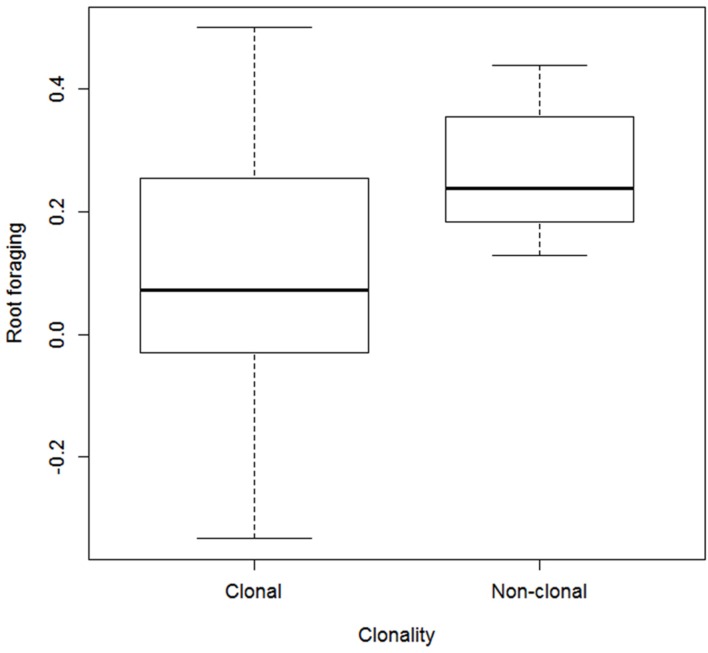
**Foraging estimates in clonal vs. non-clonal species.** Widths of the boxes show square-root of number of cases (species).

Root foraging ability substantially negatively correlated with vegetative reproduction potential (**Figure 3**) and plant clonality (binary trait) (**Table 1**). The correlation was negative; that is, clonal plants responded less (**Figure 4**). Results were approximately the same regardless of the form of reaction (directionless or directed, i.e., plasticity or foraging) used. The only exception was correlation of root allocation with lateral spread, which showed almost no correlation with the directed response (“foraging”, i.e., including rich patch avoidance as a negative response) whereas it showed much higher correlation with “plasticity” (i.e., directionless measure): (clonal) plants with low lateral spread tend to respond to soil heterogeneity level much more than plants with high lateral spread. We did not detect any other substantial correlation between root foraging or plasticity and the other traits. The results did not qualitatively change in phylogenetic analyzes (data now shown).

**Table 1:** Correlation of foraging response and species traits.

	SLA [m^2^/g] (r)	log Leaf area [m^2^] (r)	Height at maturity [m] (r)	Lateral spread [m] (r)	Generative reproduction potential [1..5] (ρ)	Vegetative reproduction potential [1..5] (ρ)	Shoot lifespan [years; 1/2] (ρ)	Clonality [0/1] (ρ)
**FORAGING**								
r/ρ	-0.03	0.24	-0.25	-0.1	0.17	-0.41	0.19	-0.48
N	33	31	37	22	35	37	37	37
P	0.862	0.191	0.133	0.648	0.331	0.012	0.256	0.003
**PLASTICITY**								
r/ρ	-0.03	0.2	-0.23	-0.34	0.17	-0.42	0.08	-0.35
n	33	31	37	22	35	37	37	37
p	0.889	0.28	0.177	0.118	0.334	0.01	0.62	0.035


*Foraging response either included the direction of the response (part FORAGING) or just its (directionless) magnitude (PLASTICITY). Type of the correlation coefficient used is indicated columnwise, r – Pearson’s correlation coefficient, ρ – Spearman’s correlation coefficient (rho). *n* – number of cases (species), *p* – *p*-value.*

## Discussion

We showed that root foraging is apparent and that plant species strongly differ in their root foraging ability – this is in accord with previous studies. We interpret root foraging mainly as an outcome of active phenotypic plasticity, that is, plant behavior. The main piece of evidence comes from the fact that the plants with the most intensive vegetative growth foraged least, as predicted if the active plasticity was the driver of the process. We show that root foraging is much less pronounced in plant species capable of vigorous vegetative growth and reproduction, that is, clonal plants. Importantly, we found no relationship between species traits used as proxies of growth rate (i.e., leaf economy traits) and root foraging when the latter was expressed using a size-invariant measure. Therefore, we assume that such relationships, reported in a previous study (Keser et al., 2014), are passive plasticity effects caused by simple differences in size across species (Aanderud et al., 2003).

The difference between clonal and non-clonal plants is the strongest pattern in root plasticity found in the experiment. This is, to our knowledge, the first systematic report of such a difference between clonal and non-clonal plants, although both Campbell et al. (1991) and Kembel and Cahill (2005) point in the same direction. This also suggests that plant clonality may be an important trait missing from the analysis of plant traits related to root foraging (Kembel et al., 2008).

It is likely that the low root foraging ability of clonal plants may derive from their ability to form stands of several ramets. Low root foraging of individual ramets may thus be compensated at the level of ramets (de Kroons and Hutchings, 1995), if foraging for nutrients is needed at all. In such a scenario, instead of proliferation and elongation of costly roots, which are effective in harvest but less so in transport (Alpert and Mooney, 1986), an entirely new semi-autonomous harvesting unit (ramet) may be deployed at the resource-rich patch, with a stem-derived spacer capable of high-capacity transport. Such transport is much more efficient than the transport efficiency of roots, so it allows specialization among ramets, that is, division of labor (Stuefer et al., 1996). On the other hand, we did not limit ramet development (by any other means than by the length of the experiment), so either we see demonstration of root placement according to future plans for ramet placement (de Kroon and Schieving, 1990; Huber et al., 1999; Holzapfel and Alpert, 2003; Gruntman and Novoplansky, 2004; Herben and Novoplansky, 2007) or a lack of root foraging ability of a single ramet for an unknown reason. However, root system plasticity in clonal plants has been repeatedly demonstrated (Hutchings and de Kroon, 1994; Holzapfel and Alpert, 2003; Gruntman and Novoplansky, 2004; Semchenko et al., 2007), so we are more likely to expect purpose than inability of the behavior.

In theory, lack of root preference for nutrient-rich patches may also result from the lower nutrient optima of clonal plants (Groenendael et al., 1996). We do not expect this to be the mechanism underlying the observed root foraging pattern. If it were so, we should have observed strong differences between directionless and directed response, which was not the case. In a similar vein, we are not aware of any evidence that herbivores affect roots of clonal and non-clonal plants differently, what may be also the case for selection of non-foraging behavior (Tsunoda et al., 2014).

In any ecological setting, the effects of the species traits are multiplied by growth, and the modification becomes stronger with greater growth differences. We therefore believe that differences in growth rates among species are the basis of previously reported strong correlations between root foraging ability and growth rate or leaf economy spectrum (Grime et al., 1997; Aanderud et al., 2003; Kembel et al., 2008). These differences may also underlie the tight correlation between above-ground and below-ground plasticity (Campbell et al., 1991), although such correlation may also arise due to functional links between aboveground and belowground resource-acquiring organs (Freschet et al., 2015). Growth modifies active plasticity effects, either weakening or enhancing them. Furthermore, because of allometry in growth (Weiner, 2004), some species traits that we did not find to be correlated with root foraging ability may become related to root foraging in certain environments, provided these environments constrain or favor growth rate (Grime, 1977).

In a similar vein, it may be noted that our data support the idea of scale - precision trade-off in root foraging (Campbell et al., 1991; Kembel and Cahill, 2005), since we found negative correlation of vegetative growth capacity and root foraging precision. However, this could be only justified if vegetative growth capacity, strongly linked to plant clonality, reflects root foraging scale. For example, Wijesinghe and Hutchings (1997) show low precision of ramet foraging in *Glechoma hederacea* when the patch is small. While *G. hederacea* is capable of rapid growth in culture or at the sites without any other competing species, it is not the most productive or dominant species in the species pool.

Our data do not show any indication of the phylogenetic signal in the root foraging or root plasticity. Kembel and Cahill (2005) found significant, albeit not very strong, phylogenetic signal in root foraging precision in a meta-analytical study. However, as they themselves note, their study covers a proportionately large number of grasses, which showed low foraging precision. Apart of annuals, most grasses are clonal (clonality is an universal trait in the monocotyledonous clade) and therefore bias the comparison, which becomes largely comparison between clonal grasses and (often) non-clonal dicots. Absence of the phylogenetic signal in our study arises due to a much more balanced species selection, which covers both clonal and non-clonal species, and explicit use of clonality as a predictor.

Regardless of the relative significance of active or passive plasticity in the root foraging process, it may be useful to quickly identify species capable and not capable of root foraging, for example, in community assembly research. However, traits (e.g., relative growth rate, shoot plasticity, leaf economy) that have been previously linked to root plasticity (Grime et al., 1997; Kembel et al., 2008) are rather derived, with strong links to plant physiology and its potential niches. Such traits, even though linked to proximate mechanisms in the life of the individual, are difficult to obtain without the necessary burden of direct estimation. For large-scale studies, this burden may render these traits unusable. Herein lies the strength of our study: Albeit in a correlative way, we identified easy-to-obtain species traits that can serve as a proxy of the plastic response including its active component. Moreover, if species coexistence is interaction driven, traits that describe the interactive interface of the individuals may be more important than the inner, non-manifested traits.

As usual for comparative studies, several caveats should be noted. First, phenotypic plasticity in the strictest sense is only detectable comparing individuals of the same genotype, and genotypes may vary in the amount of phenotypic plasticity they allow. However, since genotypes within species are more related than genotypes across species, phenotypic plasticity at the species level seems to be a reasonable estimate when resources are too limited to involve good number of replicates per genotype per species. Second, similar objections can be raised against using trait data mined from (although well established and apparently reliable) databases. Database data represent “mean potential” of the species, although particular genotype or particular experimental treatment may exhibit different values. Third, even though most of the plants did not resemble seedlings at all, they were harvested young given that species examined were perennial, and, maybe even more importantly, they were harvested in a single point of time. It is quite possible that, e.g., new clonal ramets will be deployed at the rich patches later, so the clonal foraging became apparent, or that foraging plants would later produce more roots also in poor patches just to use the tiny surplus of nutrients the poor patches provided. Temporal variation in root placement may be an adaptation to belowground competition (Hodge et al., 1999; Craine et al., 2005; Trinder et al., 2012).

## Conclusion

Simple life-history species traits (namely, potential for vegetative growth and reproduction, clonality) seem to be good correlates of root foraging as a form of active behavior. The unexpected effect of clonality on root foraging may shed new light on our understanding of clonal species growth patterns in response to resource availability and spatial heterogeneity (de Kroon and Schieving, 1990; Oborny et al., 2012). This is likely to be another example of deep functional differences between clonal and non-clonal species (Herben et al., 2015; Klimešová et al., 2016).

Further, our findings shed new light on the existing reports that plasticity may contribute to the ecological success of species. This has been reported for plasticity in root placement (Keser et al., 2015) and a similar finding has been reported for plastic response to light, where species with better plastic response are likely to occur in more species-rich communities (Lepik et al., 2005). These reports imply that the ability for plastic response is involved in community-level processes, such as patterns of species abundance and coexistence. Here we show that plasticity in root placement is, to some extent, predictable by easily obtainable traits, although the generality of this finding remains to be determined. Such traits can thus be used in much wider analyzes of patterns of species abundance and coexistence together with other species (soft) traits.

## Author Contributions

MW analyzed the data and wrote the manuscript; MW and TK carried out the experimental work; TH and TK helped draft the manuscript. All authors designed the study and interpreted the results. All authors gave final approval for publication.

## Conflict of Interest Statement

The authors declare that the research was conducted in the absence of any commercial or financial relationships that could be construed as a potential conflict of interest.

## Footnotes

1 http://plantanaturalis.com

## Supplementary Material

**TABLE S1 | Species list and pot counts.**

**TABLE S2 | Correlation coefficients of species traits.**

**FIGURE S1 | Bootstrapped species response to contrast.**

**FIGURE S2 | Species foraging and clonality mapped on the phylogenetic tree.**
